# Inhibition of TIR Domain Signaling by TcpC: MyD88-Dependent and Independent Effects on *Escherichia coli* Virulence

**DOI:** 10.1371/journal.ppat.1001120

**Published:** 2010-09-23

**Authors:** Manisha Yadav, Jingyao Zhang, Hans Fischer, Wen Huang, Nataliya Lutay, Christine Cirl, Josephine Lum, Thomas Miethke, Catharina Svanborg

**Affiliations:** 1 Department of Microbiology, Immunology and Glycobiology (MIG), Institute of Laboratory Medicine, Lund University, Sweden; 2 Singapore Immunology Network (SIgN), Biomedical Sciences Institutes, Agency for Science, Technology, and Research (A*STAR), Immunos, BIOPOLIS, Singapore, Singapore; 3 Institut für Medizinische Mikrobiologie, Immunologie und Hygiene, Technische Universität München, München, Germany; Stanford University, United States of America

## Abstract

Toll-like receptor signaling requires functional Toll/interleukin-1 (IL-1) receptor (TIR) domains to activate innate immunity. By producing TIR homologous proteins, microbes inhibit host response induction and improve their own survival. The TIR homologous protein TcpC was recently identified as a virulence factor in uropathogenic *Escherichia coli (E. coli)*, suppressing innate immunity by binding to MyD88. This study examined how the host MyD88 genotype modifies the *in vivo* effects of TcpC and whether additional, TIR-domain containing proteins might be targeted by TcpC. In wild type mice (wt), TcpC enhanced bacterial virulence, increased acute mortality, bacterial persistence and tissue damage after infection with *E. coli* CFT073 (TcpC+), compared to a Δ*TcpC* deletion mutant. These effects were attenuated in *Myd88^−/−^* and *Tlr4^−/−^* mice. Transcriptomic analysis confirmed that TcpC inhibits MYD88 dependent gene expression in CFT073 infected human uroepithelial cells but in addition the inhibitory effect included targets in the TRIF and IL-6/IL-1 signaling pathways, where MYD88 dependent and independent signaling may converge. The effects of TcpC on bacterial persistence were attenuated in *Trif ^−/−^* or *Il-1β ^−/−^* mice and innate immune responses to Δ*TcpC* were increased, confirming that Trif and *Il-1β* dependent targets might be involved *in vivo,* in addition to Myd88. Furthermore, soluble TcpC inhibited Myd88 and Trif dependent TLR signaling in murine macrophages. Our results suggest that TcpC may promote UTI-associated pathology broadly, through inhibition of TIR domain signaling and downstream pathways. Dysregulation of the host response by microbial TcpC thus appears to impair the protective effects of innate immunity, while promoting inflammation and tissue damage.

## Author Summary

The clinical manifestations of infection range from beneficial, asymptomatic states to life threatening disease, depending on the arsenal of virulence factors carried by the bacteria and the host immune defence repertoire. Pathogenic bacteria have evolved many sophisticated ways of avoiding the host defence and especially the immune response to infection. In this study, we present a very interesting case where bacteria actively inhibit the immune response by producing a host defence like protein, TcpC, which acts by promoting bacterial survival and corrupting the tissue response to infection such that the tissues are damaged rather than protected. The importance of TcpC is demonstrated in a mouse model of urinary tract infection (UTI) and in isolated human and murine kidney cells. The results suggest that TcpC expressing bacteria cause death, inflammation and tissue damage in normal hosts by creating a dysfunctional innate immune response and that partial inhibition of adaptor proteins turns the normally protective defense into lethal inflammation, followed by kidney tissue damage. In human cells, TcpC was a broad innate immune inhibitor, acting via the MYD88, TRIF and IL-1/IL-6 pathways. Our report increases the understanding of how TcpC and microbial proteins with similar targets succeed in shifting the balance in favor of the pathogen, thus promoting disease. These data are fundamentally important in showing pathways for host defense that can be fine tuned by a bacterial virulence factor in order to paradoxically promote bacterial replication thereby illustrating the host response as a generator of pathology.

## Introduction

Toll-like receptors (TLRs) control innate host responses to mucosal and systemic infections and signaling involves the intracellular Toll/interleukin-1 receptor (TIR) domain [1]. Following ligand binding, signaling is initiated by the recruitment of adaptor proteins to the TIR domain [2], [3], [4], including myeloid differentiation factor-88 (MYD88), MYD88 adapter-like protein (Mal), TIR domain-containing adaptor protein inducing IFNβ (TRIF), TRIF-related adaptor molecule (TRAM) and the sterile α- and armadillo-motif-containing protein (SARM). Negative regulators of TLR signaling include SIGIRR, MyD88s and IRAK-M, which block MyD88 dependent activation, or Triad3A and SARM, which block the TRIF dependent pathway. The SIGIRR TIR domain resembles MyD88 but lacks two amino acids needed for signaling to occur [5], [6]. However, TIR-TIR interactions between SIGIRR and TLR4 prevent the recruitment of IRAK and TRAF6 to MyD88 [6]. MyD88s is a splice variant inhibiting MyD88 dependent TLR4 activation by allowing MyD88 to bind the intermediate IRAK-binding domain without inducing IRAK phosphorylation and NF-κB activation [7]. IRAK-M prevents IRAK and IRAK-4 dissociation from MyD88 and TRAF6 complex formation [8]; Triad3A interacts with TIR domains of TLRs, TRIF, TIRAP and RIP1 [9]; and SARM blocks gene induction downstream of TRIF [10]. Competition at the level of the TIR domain is thus used by host cells to modify TLR signaling in response to specific agonists [6], [7], [11], [12].

Pathogens have also evolved mechanisms to inhibit the TLR dependent host defense and to increase their fitness and virulence for a specific host niche [12]. The TIR domain plays a crucial role in the mammalian innate immune response and recently proteins containing TIR domains have been described in a wide variety of bacteria, fungi, archaea and viruses [13]. Whole genome sequencing and structural studies have revealed that several pathogens carry TIR-domain homologous sequences, including two proteins from Vaccinia virus A46R and A52R, which interfere with IL-1 and TLR4 mediated activation of NF-κB [14]. Similar proteins were identified in *Salmonella*, *Brucella* and uropathogenic *E. coli* (UPEC) [12], [15], [16]. On the other hand, Spear and co-workers suggested that most TIR domains in bacteria did not evolve to subvert the function of eukaryotic cells but simply to function as general purpose protein-protein interaction domains for diverse uses [13]. We recently showed that the TIR homologous protein TcpC in the UPEC strain *E. coli* CFT073 acts as a virulence factor by suppressing innate host responses in the kidney, enhancing bacterial persistence and tissue damage [12]. Epidemiologic studies of patient *E. coli* isolates showed that TcpC occurred more frequently in strains causing severe kidney infections than in *E. coli* causing other forms of urinary tract infection (UTI) [12]. The UTI model is therefore quite suitable to investigate the mechanisms of TcpC-modulation of the innate host response *in vivo* and the consequences for bacterial persistence and disease severity [11].

TcpC binds to MyD88 [12] but it remains unknown if other TIR-domain containing molecules of the host are influenced by TcpC *in vivo*. This study addressed this question with the aim to define the genetic control of TcpC mediated immune inhibition *in vivo.* We used the murine UTI model and mice lacking specific innate immune response genes to examine whether Myd88 controls the TcpC dependent response to UPEC infection and if additional Myd88 independent signaling pathways might modify the effects of TcpC *in vivo*. Furthermore, transcriptomic and proteomic analysis of infected human epithelial cells was used to define targets of TcpC and effects on innate immune activation by UPEC and inhibition of TLR signaling by soluble TcpC protein was defined in murine macrophages. The results suggest that TcpC partially inhibits TIR domain dependent signaling *in vivo* and in host cells including pathways downstream of MYD88, where MYD88 dependent and independent innate immune responses may converge. In this way, broad but incomplete suppression by microbial TcpC, may impair innate immune protection, while promoting inflammation and tissue damage.

## Results

### TcpC increases bacterial burden, abscess formation and tissue pathology

**Figure 1:**
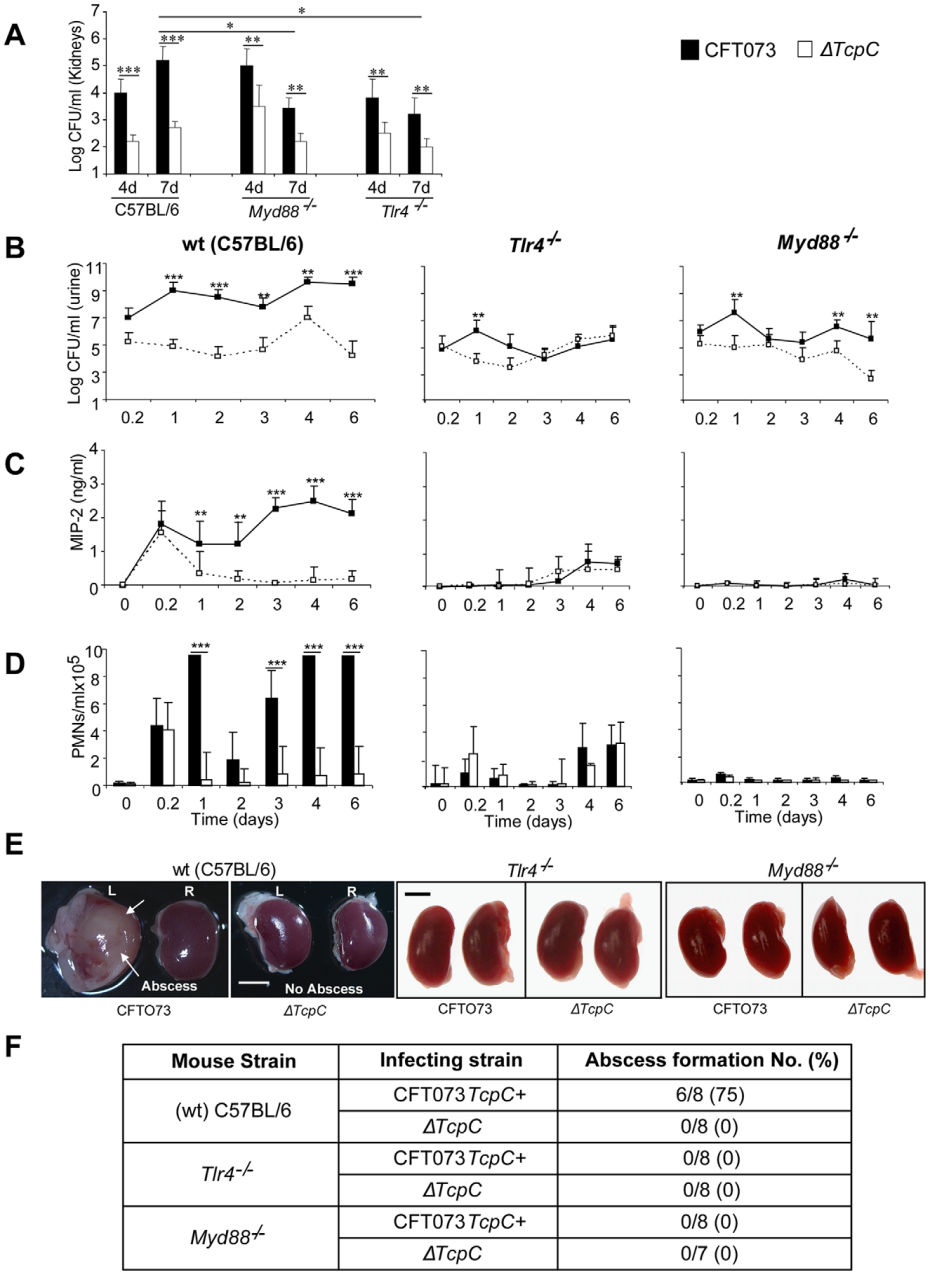
TcpC acts as a virulence factor by promoting bacterial persistence in the urinary tract and abscess formation in the kidneys of wild type mice. (A) Bacterial counts (CFUs) in kidneys of C57BL/6, *Tlr4^−/−^* and *Myd88^−/−^* on days 4 and 7 post-infection. (B) Bacterial counts (CFUs) in urine of C57BL/6, *Tlr4^−/−^* and *Myd88^−/−^* mice after infection with CFT073 or Δ*TcpC.* (C) MIP-2 response kinetics in urine samples of wt and mutant mice. (D) Neutrophil response kinetics in urine samples of wt and mutant mice. P values (*p<0.05, **p<0.01 and ***p<0.001 for CFT073 versus Δ*TcpC* mutant and for wt versus mutant mice; Fisher's exact test). Eight to ten mice were used per time point. (E) Abscess formation (arrows) in the kidneys of wt mice infected with CFT073 but not in *Tlr4^−/−^* and *Myd88^−/−^* mice infected with CFT073 or Δ*TcpC* (Scale bar, 2 mm).(F) Abscess formation after CFT073 or Δ*TcpC* infection, in percent of the total number of kidneys examined.

Experimental UTI was established in wild type (wt) C57BL/6 mice by intravesical infection with *E. coli* CFT073 (CFT073) or the CFT073*tcpC::kan* mutant (Δ*TcpC*) [12] and was monitored for seven days. A higher bacterial burden was present after CFT073 infection compared to Δ*TcpC* (p<0.001, Figure 1B) in urine samples obtained daily after infection. This difference was reproduced in kidneys (p<0.001) obtained on days four or seven after infection (Figure 1A). MIP-2 chemokine concentrations and neutrophil responses in urine were higher in mice infected with CFT073 compared to Δ*TcpC* (p<0.001; Figure 1C, D). Tissue damage was extensive in mice infected with CFT073; the kidneys were large, pale and soft and abscesses were present in 75% of the organs (Figure 1E, F). In contrast, kidneys of mice infected with Δ*TcpC* were normal in size, color and texture and lacked detectable abscesses (p<0.05, Figure 1E, F).

### The host Tlr4 and Myd88 genotypes control CFT073 and Δ*TcpC* infection and renal pathology

Experimental UTI with CFT073 or the Δ*TcpC* mutant was subsequently established in Tlr4 and Myd88 adaptor protein knockout mice in the C57BL/6 background (*Tlr4^−/−^* and *Myd88^−/−^* respectively). Total bacterial counts and the TcpC dependent difference in bacterial persistence were reduced in the mutant mice, compared to wt mice (Figure 1). Bacterial counts in urine were lower in *Myd88^−/−^* (p<0.05) and *Tlr4^−/−^* (p<0.05) mice as compared to wt mice (Figure 1). In addition, the renal bacterial counts were significantly lower in mutant mice on day seven than in wt animals which were infected with CFT073 (p<0.05, Figure 1A). However, there was no difference in bacterial counts in wt and mutant mice which were infected with Δ*TcpC* (Figure 1A). The MIP-2 and neutrophil responses were absent in infected *Myd88^−/−^* mice and drastically reduced in *Tlr4^−/−^* compared to C57BL/6 wt mice (p<0.0001, Figure 1C, D), confirming that these aspects of the early innate immune response require Myd88 and Tlr4 dependent signaling. Kidneys of *Tlr4^−/−^* and *Myd88^−/−^* mice infected with CFT073 or the Δ*TcpC* mutant were normal in size, color and texture and had no abscesses (Figure 1E, F).

TcpC-related differences in bacterial persistence were observed also in the mutant mice (Figure 1). *Tlr4^−/−^* and *Myd88^−/−^* mice developed significant bacteriuria (≥10^5^ CFU/ml of urine) six hours after infection with CFT073 or Δ*TcpC* and bacteria persisted in urine until the experimental end point. Higher numbers of CFT073 than Δ*TcpC* were observed on day one in *Tlr4^−/−^* mice (p<0.01); and on days one, four and six in *Myd88^−/−^* mice (p<0.01, Figure 1B). In addition, CFT073 numbers were higher than Δ*TcpC* in kidneys of *Myd88^−/−^* and *Tlr4^−/−^* mice (p<0.01) on days four and seven (Figure 1A). The difference between CFT073 and Δ*TcpC* was reduced compared to wt mice in *Tlr4^−/−^* (p<0.01) and *Myd88^−/−^* mice (p<0.01) by more than two logs in urine (Figure 1A). TcpC dependent increases in MIP-2 and neutrophil responses observed in wt mice were lost in *Tlr4^−/−^* mice, the, confirming the essential role of TLR4 and its TIR domain for innate immune responses to UTI (Figure 1C and D). The *Myd88^−/−^* mice did not mount MIP-2 and neutrophil responses to CFT073 or ΔTcpC infection. These results suggest that TcpC affects bacterial persistence and pathology, in part, via Tlr4 and Myd88 dependent but also via Myd88 independent pathways.

### Absence of kidney pathology in *Tlr4* and *Myd88* knockout mice

**Figure 2:**
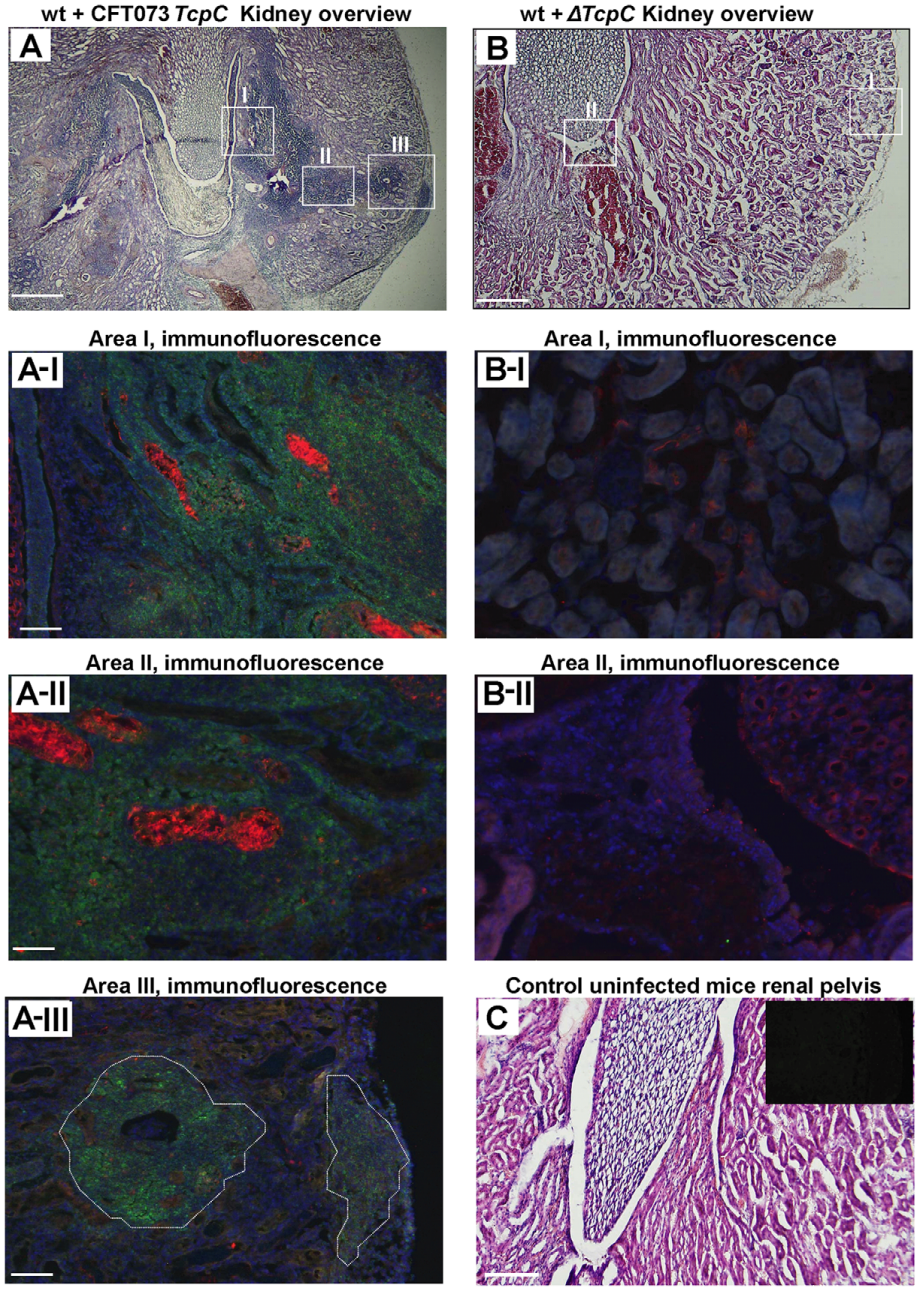
Deletion of TcpC abrogates abscess formation in kidney sections from wild type mice infected with CFT073. (A) Overview of htx-eosin stained kidney section of wt mice infected with CFT073, showing abscesses (scale bar, 500 µm). Magnified areas of section A shown as A-I, A-II and A-III are stained with specific antibodies to neutrophils (green, NIMP-R14) and the PapG adhesin (red, antiserum to a synthetic PapG peptide) (scale bar, 100 µm). Abscesses in wt mice are shown by dotted lines. (B) Overview of kidney section of wt mice infected with Δ*TcpC* (scale bar, 300 µm). Magnified areas of section B shown as areas B-I and B-II are stained with specific antibodies as described above. (C) Kidney section of uninfected control mice with htx-eosin staining (scale bar, 200 µm) and inset picture showing negative control for anti-neutrophil and anti-PapG antibodies.

Kidney sections from CFT073 infected wt mice (htx-eosin, day 7) showed swollen collecting ducts and inflammatory cell infiltrates into the kidney parenchyma (Figure 2A). P-fimbriated bacteria were present in the tissues, from the pelvic region to the cortex, as shown by PapG specific antibody staining. Neutrophils were abundant in the abscesses and scattered throughout the tissue, as shown by a neutrophil specific antibody (areas AI-AIII in Figure 2A). By dual staining, P fimbriae and neutrophils were detected in the abscesses and collecting ducts (Figure 2A). In contrast, kidney sections from mice infected with Δ*TcpC* showed normal structure, no bacteria and few inflammatory cells (areas BI-BII in Figure 2B). Figure 2C shows htx-eosin stained sections from an uninfected control kidney and the inset shows a negative control section stained with the anti-neutrophil and anti-PapG antibodies. The results suggest that TcpC reduces host resistance and increases inflammation, resulting in a high bacterial burden and tissue damage as these sequels become attenuated in mice infected with the Δ*TcpC* mutant.

**Figure 3:**
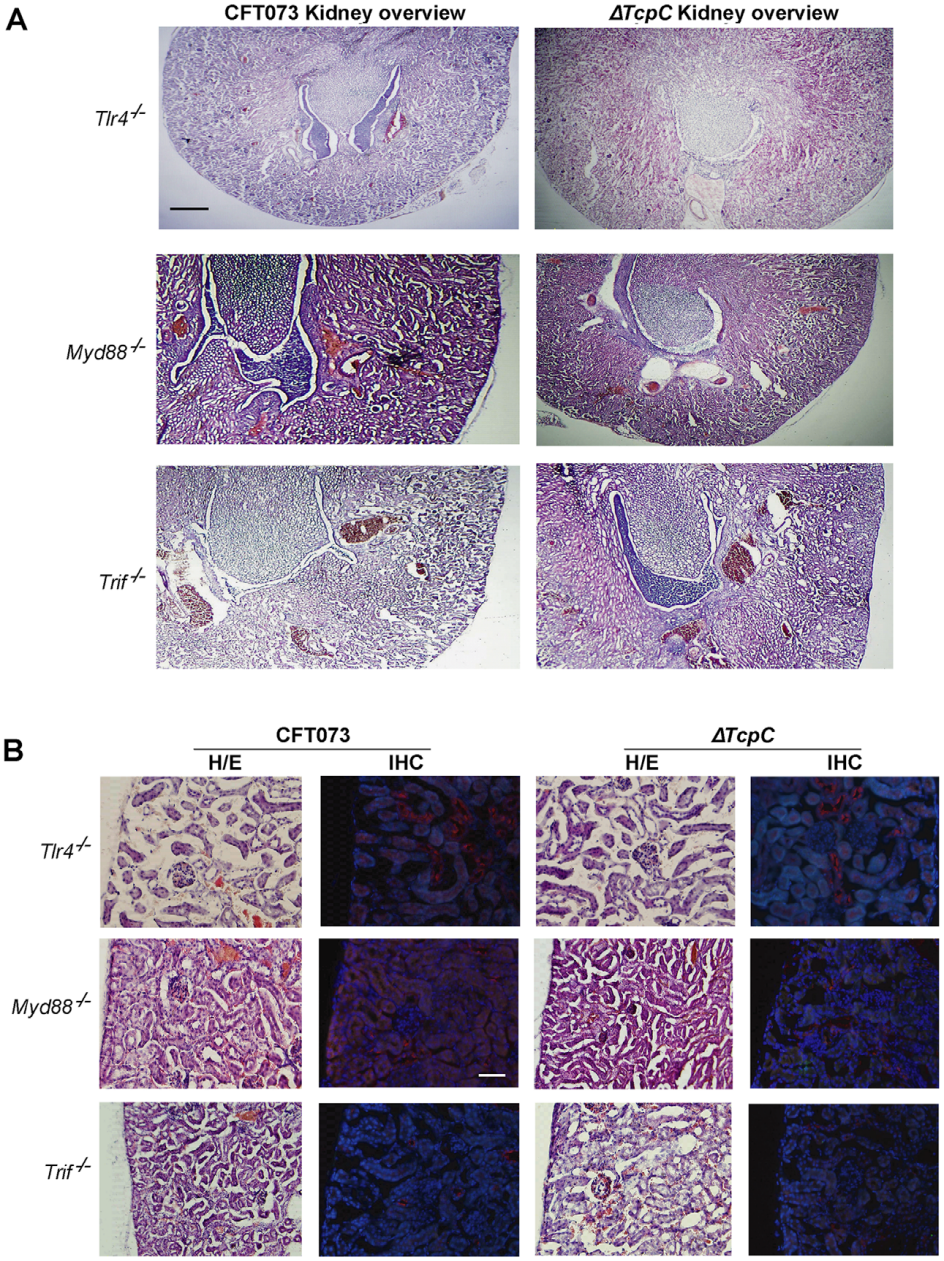
Morphology of intact kidney tissue in *Tlr4 ^−/−^* and adaptor protein mutant mice infected with CFT073 and Δ*TcpC*. (A) Overview of htx-eosin stained kidney section of *Tlr4^−/−^*, *Myd88^−/−^* and *Trif ^−/−^* mice. Scale bar, 500 µm. (B) Histology of renal cortex stained with specific antibodies to neutrophils (green, NIMP-R14) or the PapG adhesion (red, antiserum to a synthetic PapG peptide). Scale bar, 100 µm. Neutrophils or bacteria were not detected in the tissues. The *Tlr4^−/−^* and *Myd88^−/−^* mice were compared to C57BL/6 wt mice and *Trif ^−/−^* mice to C57BL6/129 wt mice.

Htx-eosin stained sections from *Myd88^−/−^* and *Tlr4^−/−^* mice infected with CFT073 showed normal collecting ducts, few inflammatory cells and no bacteria in the medulla or cortex of kidneys from either host (Figure 3A). P fimbriae or neutrophils were not detected in infected kidneys by immunohistochemistry (Figure 3B). Similarly, there was no tissue pathology in *Myd88^−/−^* and *Tlr4^−/−^* mice infected with the Δ*TcpC* mutant. The results suggest that a host response involving Tlr4 and Myd88 leads to TcpC dependent kidney pathology after CFT073 infection and that hosts lacking Tlr4 or the adaptor are protected from such tissue damage.

### Transcriptomic analysis of host responses to CFT073 and Δ*TcpC* in human uroepithelial cells

**Figure 4:**
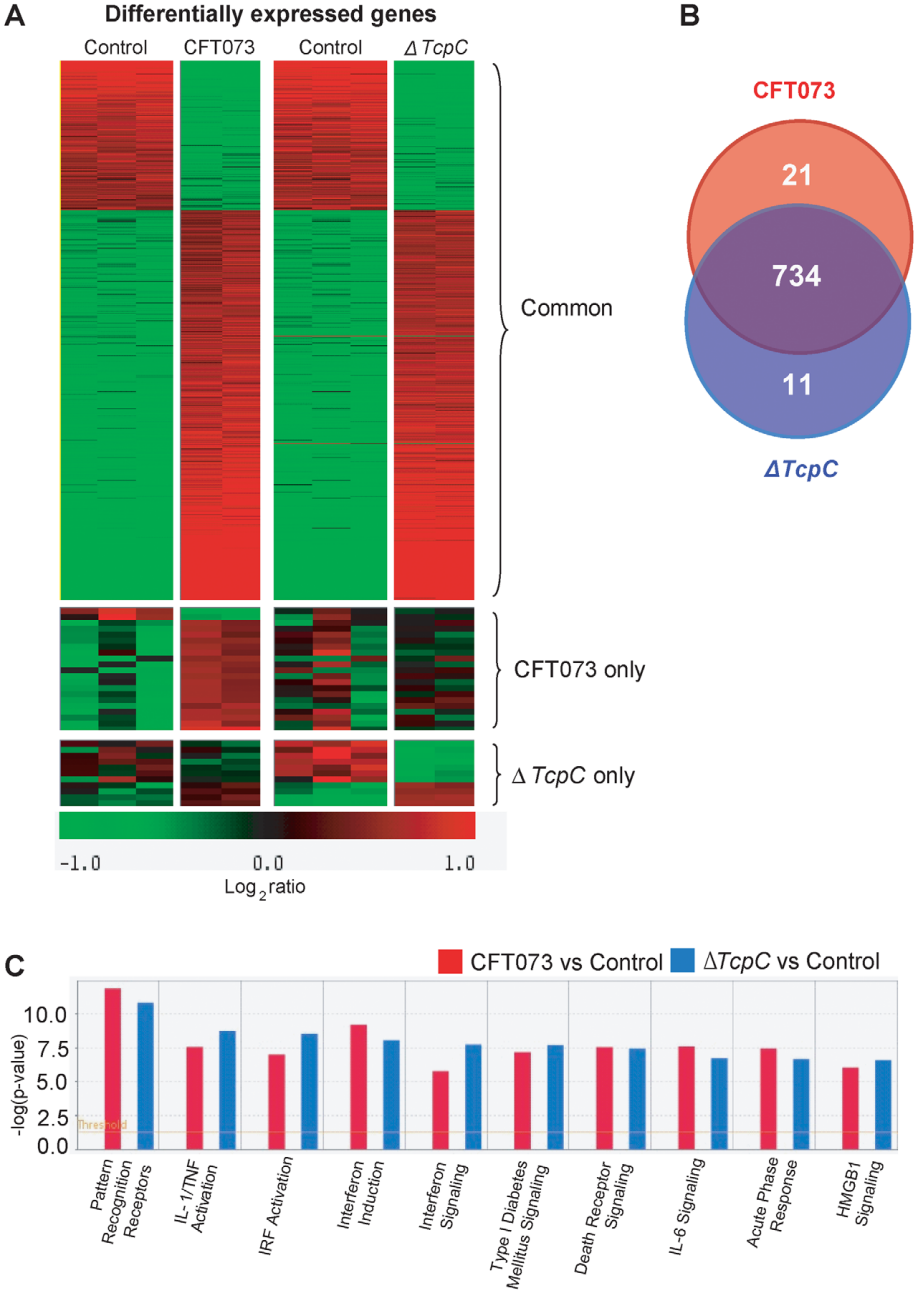
Uroepithelial gene expression in response to *in vitro* infection with CFT073 or *ΔTcpC*. (A) A two-dimensional hierarchical heat map illustrating differentially transcribed genes in human A498 cells infected with CFT073 or *ΔTcpC* versus the control group. Analysis was performed on 766 genes with a fold change of at least two over untreated cells, and classified based on whether they are CFT073 or *ΔTcpC* specific, or common to both infections. Induced genes are represented by brighter shades of red, while down regulated genes are represented by brighter shades of green (see color scale). (B) Venn diagram showing numerical distribution of differentially expressed genes in CFT073 (red) and *ΔTcpC* infected (blue) cells compared to mock infected cells. (C) A comparison of the 10 highest scoring biological pathways between infected cells and controls as analyzed by Ingenuity Pathway Analysis with default settings.

While the *in vivo* experiments confirmed that TcpC mediated virulence depends on pathways controlled by Myd88, they also suggested that TcpC modifies additional host response pathways. To identify such pathways, human A498 kidney epithelial cells were infected *in vitro* with CFT073 or Δ*TcpC* (4 hours, 10^8^ CFU/ml) and complementary RNA was hybridized to Illumina whole genome microarrays. A heat map of significantly regulated genes is shown in Figure 4A (means of triplicate spots). In identification of CFT073 or Δ*TcpC*-specific genes, fold changes of ≥2 were used. For the comparison of CFT073 or Δ*TcpC,* a fold change in response to either strain >1.5 relative to the respective control was used.

**Figure 5:**
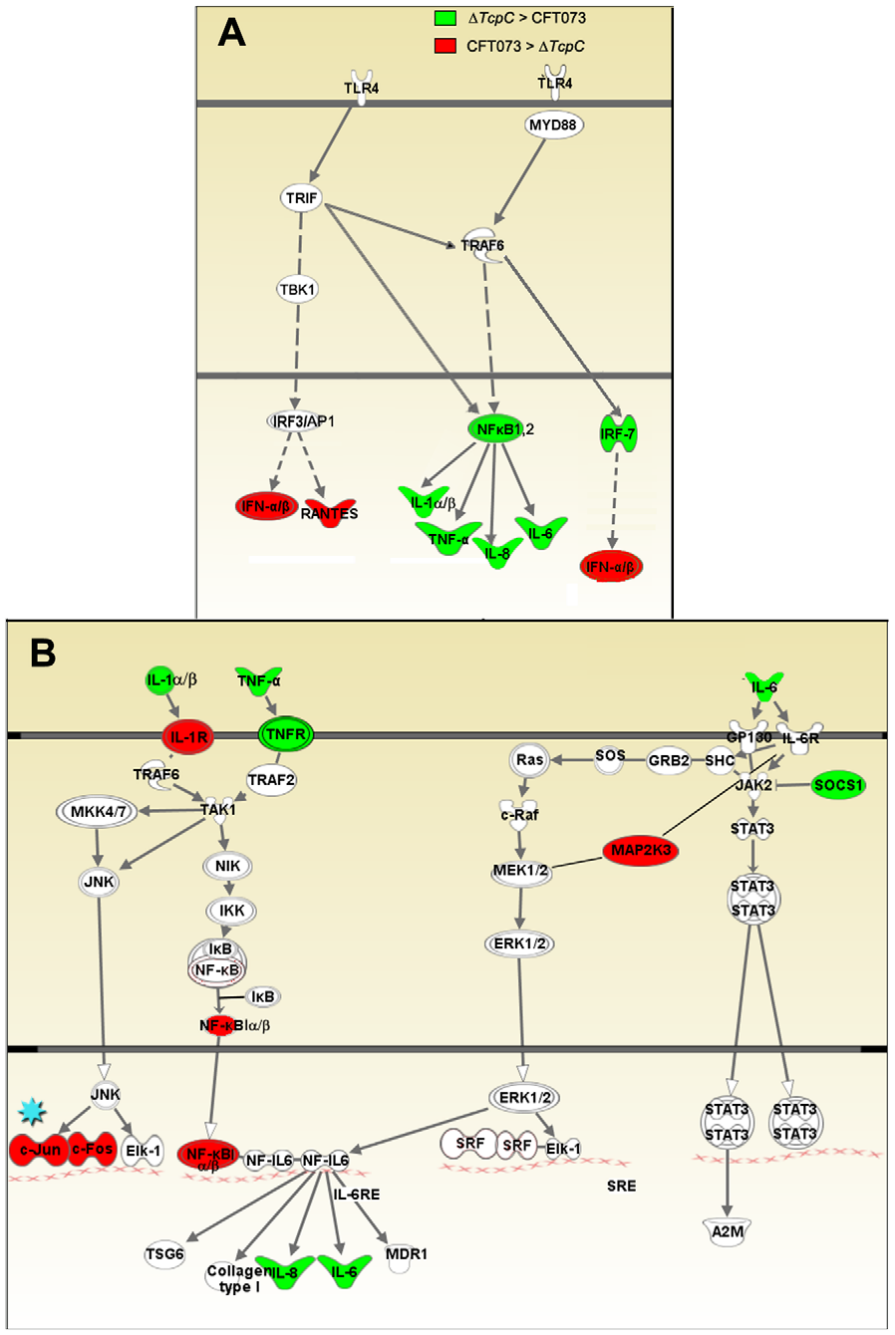
Identification of pathway-specific genes by Ingenuity Pathway Analysis in response to *in vitro* infection. (A-I) Pattern recognition pathway. (A-II) IL-6/IL-1 pathway. Each node represents a protein whose functional class is represented by various shapes (ovals = transcription factors, spiral = kinases, dumbbell = transcription regulators, V shape = cytokine or growth factors, Y shape = transmembrane receptors, circles = others). Direct protein interactions are represented by solid lines, while dashed lines indicate indirect interaction. A red node indicates up regulation of a protein in response to CFT073, while a green node indicates down regulation by CFT073 relative to *ΔTcpC*.

Infection stimulated marked changes in gene expression and three major groups of genes were altered; 734 regulated genes were shared, 21 genes responded only to CFT073, while 11 responded only to Δ*TcpC* (Figure 4B, Supplementary Table S1). Differentially expressed genes between unstimulated and CFT073 or Δ*TcpC* treated cells were then characterized using Ingenuity Pathway Analysis. Notably, signaling downstream of pattern recognition receptors, interferon induction, interferon response and IL-6/IL-1 signaling pathways were among the top-scoring pathways identified (Figure 4C). To further study the mechanisms of TcpC mediated innate immune inhibition, differentially transcribed genes were assigned to known response pathways (Figure 5, Supplementary Table S1). Consistent with the proposed role of TcpC as a MYD88 inhibitor [12], *in vitro* infection with Δ*TcpC* upregulated MYD88 dependent transcripts involved in pathogen pattern recognition, compared to CFT073 infected cells (p>0.01). These include the inflammatory cytokines IL-6, IL-8, TNF-α, IL-1α/β and the transcription factors IRF7 and NF-κB1, 2 (Figure 5A, Green). In addition, the expression of downstream pro-inflammatory genes including *IL-1*α*/β, TNFAIP6* and *TNFRSF11b* were upregulated in *ΔTcpC* compared to CFT073 infected cells (Figure 5B, Green), while the transcription of negative regulators *IL-1RA*, *NFkBIa* and *NFkBIb* was reduced, consistent with activation of the MYD88 pathway (Figure 5B, Red). Interestingly, a corresponding reduction in the MAP kinase *MAP2K3*, *JUN* and *FOS* transcripts was observed, suggesting a partial rather than complete suppression of the MYD88 pathway by TcpC (Figure 5B, Red). NF-κB and IRF7 transcript levels, which are both MYD88 and TRIF dependent, were significantly increased after *ΔTcpC* infection compared to CFT073 (p<0.01), suggesting that also TRIF-dependent signaling, might be inhibited by TcpC. Other genes downstream of TRIF maintained their activity regardless of TcpC, however.

**Figure 6:**
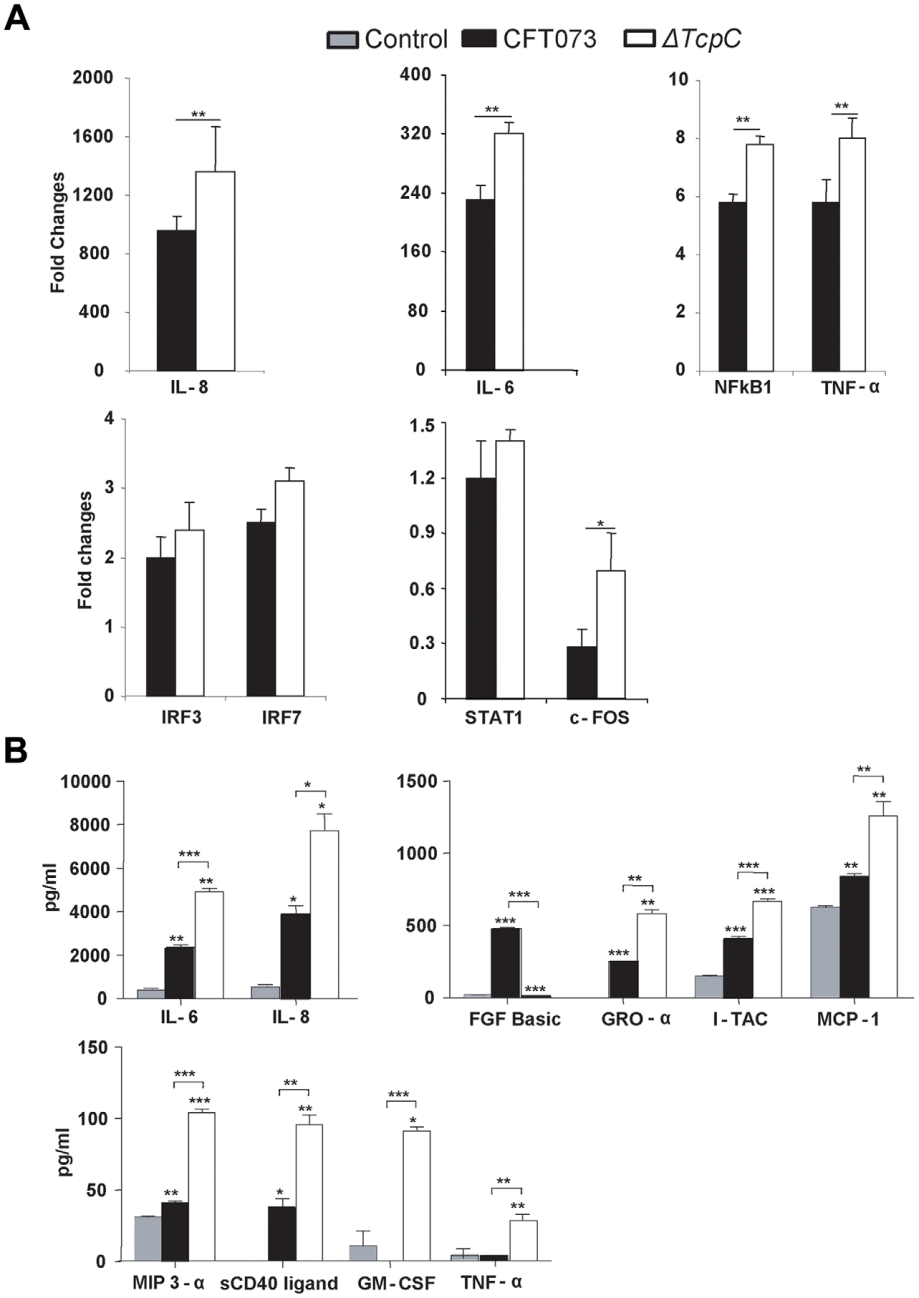
Protein array analysis in response to *in vitro* infection with CFT073 or *ΔTcpC*. (A) RT-PCR analysis of human kidney cell lines infected with CFT073 or *ΔTcpC* for 4 hrs. All values represent means ± SEMs of three experiments. (B) Uroepithelial cytokine responses to infection with CFT073, *ΔTcpC* or mock-infected controls. A498 cells were stimulated for 4 hours with the indicated bacterial strains. Supernatant cytokine levels are in pg/ml and represent means ± SEMs of three experiments. Significant differences are indicated by single * (p<0.05), double ** (p<0.01) or triple asterisks *** (p<0.001, Student's t-test).

In order to validate the transcriptomic analysis, expression levels of selected genes were confirmed by RT-PCR in the cells infected with CFT073 or *ΔTcpC* (Figure 6A). Significant differences were observed for IL-8, IL-6, NFkB1, TNF- α and c-FOS. The pattern reflected a trend similar to that revealed by microarray analysis for the gene products tested (Figure 5B*, Red).

To generate further insights into the effects of TcpC, cultured human kidney cells were stimulated with either CFT073 or *ΔTcpC* and culture medium was assayed using the Procarta human cytokine kit (Figure 6B). *ΔTcpC* stimulation resulted in significantly elevated levels of MYD88 dependent proinflammatory cytokines (IL-6, IL-8, TNF-α) (p<0.01) compared to the wild type strain, corroborating the transcriptomic analysis and suggesting that MyD88 and TRIF dependent pathways are modified by TcpC. In addition, *ΔTcpC* stimulation resulted in increased expression of the neutrophil chemoattractants MCP-1, GRO-α and MIP-3α (Figure 6B), while *in vivo* infection with *ΔTcpC* caused a lower MIP-2 response than CFT073.

The apparent discrepancies between the *in vivo data* and *in vitro* results were further examined, by establishing murine tubular kidney cell lines from wt and *Myd88* ^−/−^ or *Trif ^Lps2/Lps2^* mutant mice. The cells were infected with CFT073 or *ΔTcpC* and MIP-2 secretion was quantified by ELISA, four hours after infection (Supplementary Figure S1). MIP-2 secretion was reduced in *ΔTcpC* infected compared to CFT073 infected cells from wt mice, consistent with the increased response to CFT073 in wt mice. In *Trif ^Lps2/Lps2^* cells, MIP-2 secretion was also reduced in *ΔTcpC* compared to CFT073 infected cells (Supplementary Figure S1). Furthermore, the *in vitro* response of cells from *Myd88* ^−/−^ mice was very low, both to CFT073 and *ΔTcpC*, thus reproducing the lack of response in mutant mice. By RT-PCR, the MIP-2 response to *ΔTcpC* was reduced compared to CFT073 in cells from wt mice and also very low in cells from *Myd88* ^−/−^ mice but not different in cells from *Trif ^Lps2/Lps2^* mice (data not shown), Thus most but not all of the *in vitro* results were compatible with the *in vivo* data, either in human or murine kidney cells. Differences between *in vivo* data and *in vitro* assays are expected to occur, as the *in vivo* response of an entire organ system is unlikely to be reflected by a single cell type *in vitro*.

### TcpC dependent virulence and innate immune responses act via the Trif signaling pathway

**Figure 7:**
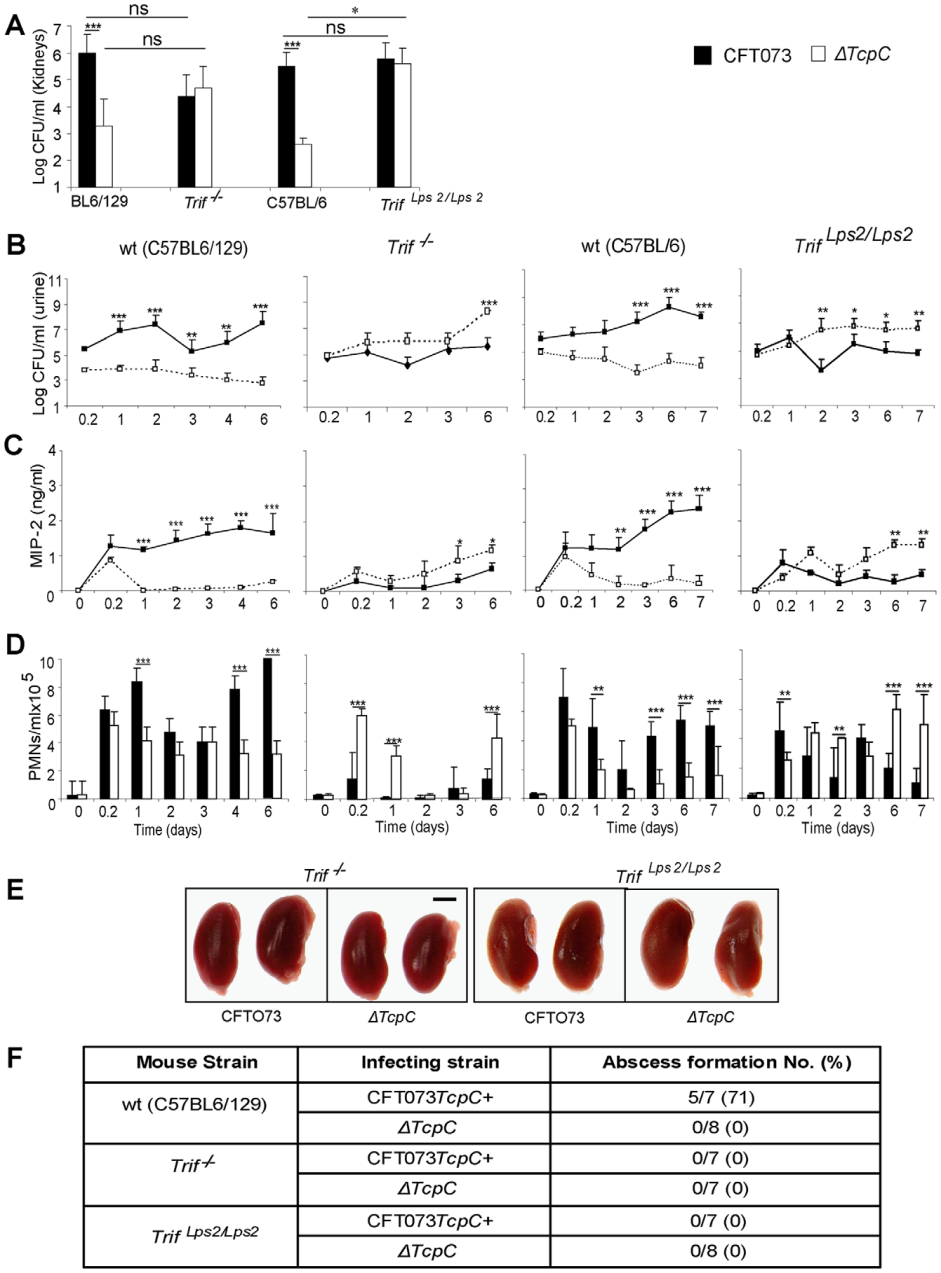
Effects of TcpC virulence in *Trif* *^−/−^ and* *Trif ^Lps2/Lps2^* mice infected with CFT073 or *ΔTcpC*. (A) Bacterial burden (CFU) in kidneys of wt (C57BL6/129), *Trif ^−/−^*, wt (C57BL/6) and *Trif ^Lps2/Lps2^* mice on day seven after infection with CFT073 or Δ*TcpC.* (B) Bacterial numbers in urine of wt and mutant mice after infection. (C) Kinetics of the MIP-2 response, measured in urine samples. (D) Neutrophil response kinetics in urine of wt and mutant mice. P values (*p<0.05, **p<0.01 and ***p<0.001 for CFT073 versus Δ*TcpC* mutant and for wt versus mutant mice; Fisher's exact test; ns = not significant). (E) Abscess formation in mouse kidneys, seven days post-infection (Scale bar, 2 mm). (F) Abscess formation after CFT073 or Δ*TcpC* infection, in percent of the total number of kidneys examined.

The cellular studies and findings in *Myd88^−/−^* and *Tlr4^−/−^* mice suggested, that bacterial TcpC might inhibit TLR4 dependent signaling, also through the Trif adaptor. To examine this hypothesis, Trif adaptor protein knockout mice (*Trif ^−/−^*) were infected with CFT073 or the Δ*TcpC* mutant and compared to wt C57BL6/129 mice. The Δ*TcpC* mutant established higher bacterial counts in urine than CFT073 in *Trif ^−/−^* mice (p<0.001), in contrast to C57BL6/129 wild type mice (Figure 7B, p<0.001). Furthermore, the difference in kidney counts between CFT073 and Δ*TcpC* in wt mice (Figure 7A, p<0.001) was not observed in *Trif ^−/−^* mice, indicating that the effects of TcpC on bacterial persistence are neutralized in mice with defective Trif signaling. *Trif ^−/−^* mice also exhibited a significant MIP-2 response to *ΔTcpC* compared to CFT073 (p<0.05, day 3 and 6) (Figure 7C) and the neutrophil response to *ΔTcpC* was significantly higher (p<0.001, Figure 7D).

The effects of Trif on TcpC inhibition were confirmed in *Trif ^Lps2/Lps2^* mice, which carry a non-functional co-dominant Trif allele, induced by N-ethyl-N-nitrosourea mutagenesis on a pure C57BL/6 background [17] (Figure 7). As in *Trif ^−/−^* mice, the Δ*TcpC* mutant established higher bacterial counts in urine than CFT073 in contrast to C57BL/6 wt mice (Figure 7B, p<0.001). The TcpC related difference in kidney counts between CFT073 and Δ*TcpC* in wt mice (Figure 7A, p<0.001) was not observed in *Trif ^Lps2/Lps2^* mice and the MIP-2 and neutrophil responses to *ΔTcpC* were significantly higher compared to CFT073 (Figure 7C, D; p<0.01). However, neither CFT073 nor *ΔTcpC* induced kidney abscesses in *Trif ^−/−^* mice or in *Trif ^Lps2/Lps2^* mice (Figure 7E, F). The results suggest that the Trif adaptor protein is involved in the innate immune mechanisms controlling the persistence of TcpC expressing bacteria.

### Inhibitory effects of TcpC on TLR signaling in murine macrophages

**Figure 8:**
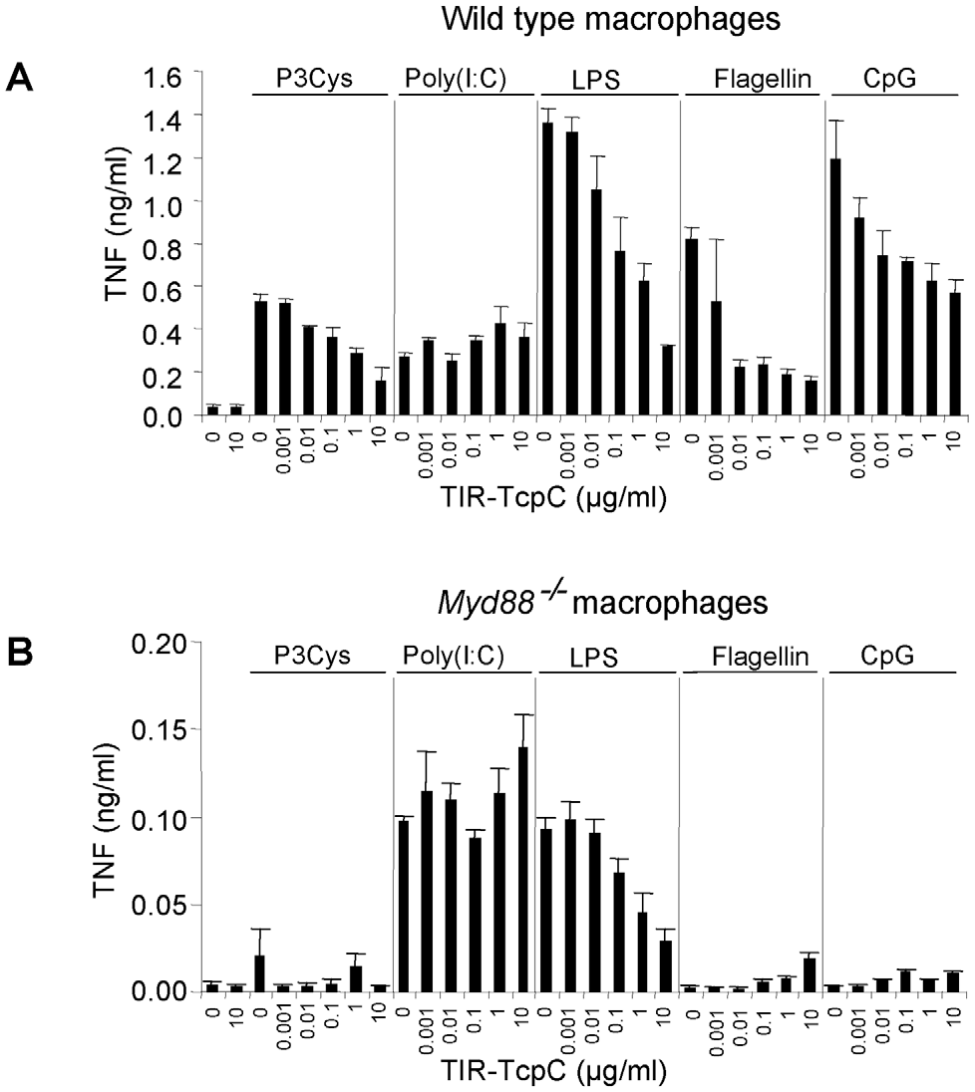
Inhibitory effects of the soluble TcpC TIR-domain on TLR signaling in murine macrophages. (A) Wt BMDM were stimulated with Pam3Cys (1 µg/ml), poly(I:C) (2.5 µg/ml), ultrapure LPS from E. coli K12 (100 ng/ml), flagellin from *S. typhimurium* (1 µg/ml) and CpG 1826 (2 µM) in the presence of titrated amounts of the purified TIR domain of TcpC (TIR-TcpC) as indicated. TNF secretion was analyzed 3 hr after stimulation. (B) The experiment in (A) was repeated at the same time, side by side with *Myd88^−/−^* BMDM. Error bars represent SD from three individual cultures. The experiment was repeated once with similar results.

To confirm that the TIR domain of TcpC impaired MyD88-dependent TLR signaling, bone marrow derived macrophages (BMDMs) from wild type or *Myd88^−/−^* mice were stimulated with different TLR ligands in the presence of titrated amounts of TIR-TcpC, the c-terminal half of TcpC containing the TIR domain. TIR-TcpC impaired TNF secretion induced by the different TLR ligands with the exception of TLR3 mediated stimulation, as expected from the MyD88 independence of TLR3 (Figure 8A) [12]. Also as expected only poly(I:C) and LPS were able to induce TNF secretion in *Myd88^−/−^* BMDMs, presumably via Trif. Interestingly, TIR-TcpC impaired this pathway as well, consistent with the *in vivo* observation that the Trif pathway was influenced by TcpC (Figure 8B). In addition, control experiments showed that TcpC is secreted into the urine of infected mice (data not shown).

### Influence of Il-1β and Irf3 on TcpC dependent virulence

**Figure 9:**
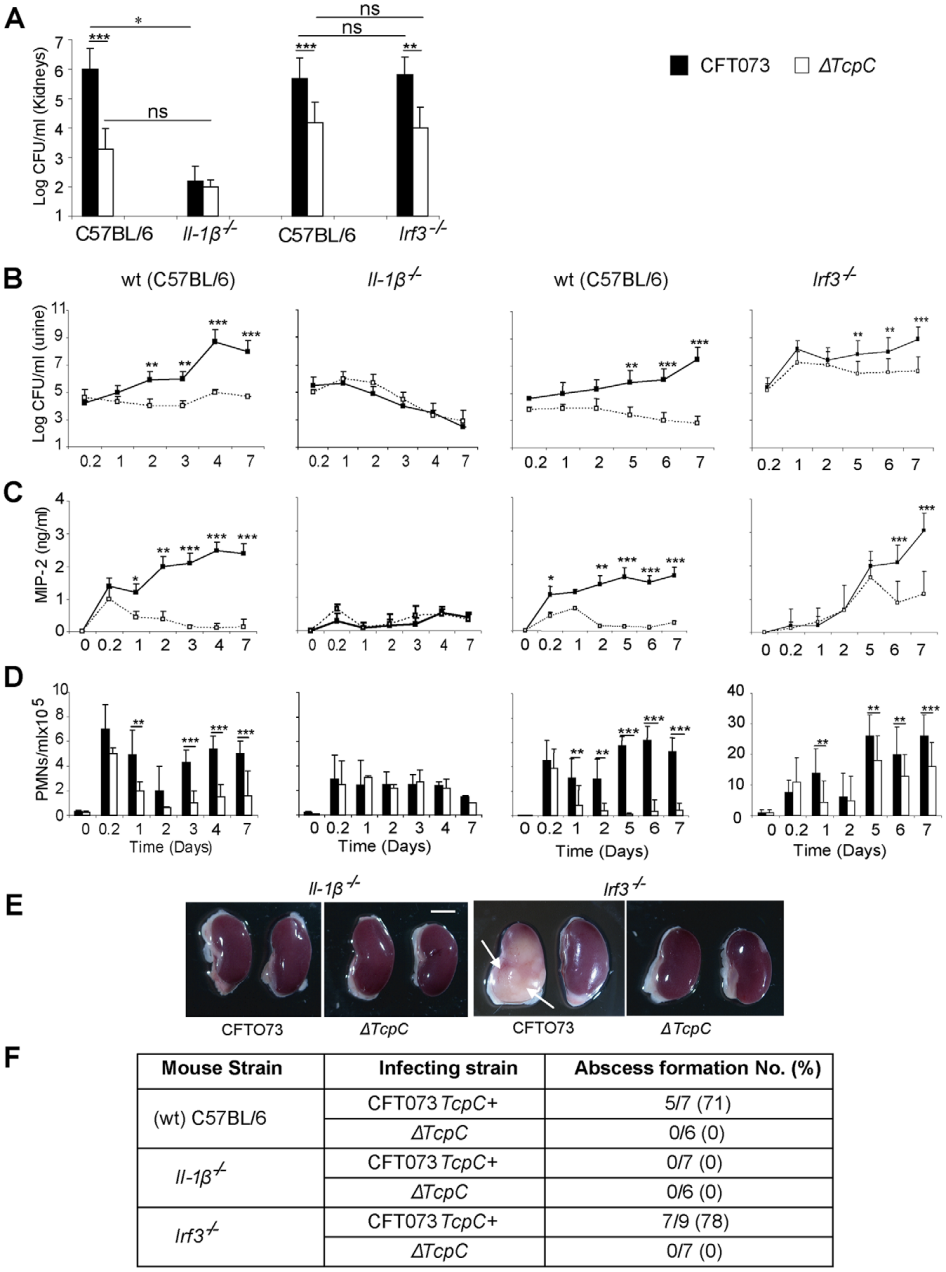
Effects of TcpC virulence in *Il-1β^−/−^ and ***Irf3^−/−^*** mice infected with CFT073 or Δ* *TcpC*. (A) Bacterial burden (CFU) in kidneys of wt (CF7BL/6), *Il-1β^−/−^* and *Irf3^−/−^* mice on day seven after infection with CFT073 or Δ*TcpC.* (B) Bacterial numbers in urine after infection. (C) Kinetics of the MIP-2 response, measured in urine samples. (D) Neutrophil response kinetics in urine of wt and mutant mice. P values (**p<0.01 and ***p<0.001 for CFT073 versus Δ*TcpC* mutant and for wt versus mutant mice; Fisher's exact test; ns = not significant). (E) Abscess formation in mouse kidneys, seven days post-infection (Scale bar, 2 mm). (F) Abscess formation after CFT073 or Δ*TcpC* infection, in percent of the total number of kidneys examined.

The transcriptomic analysis suggested that TcpC strongly regulates the pro-inflammatory cytokines IL-6 and IL-1α/β, as well as downstream signaling pathways. Enhanced expression of IL-1α/β (p<0.03 for IL-1α and p<0.02 for IL-1β) in *ΔTcpC* infected human cells suggested that the inhibitory effect of TcpC includes IL-1 dependent inflammation. To address this question, *Il-1β^−/−^* mice were infected with CFT073 or *ΔTcpC,* as described. The TcpC dependent difference in bacterial persistence and host response induction was reduced in these mice (p<0.0001, Figure 9). Renal abscess formation or tissue pathology was not observed.

IRF3 is a transcription factor controlling interferon responses to viral infection [18]. More recently, the involvement of IRFs in immunoregulation by TLRs has received more attention, since NF-kB, IRF3 and AP-1 form transcriptional complexes that regulate innate immune responses in monocytes [19]. We have recently identified IRF3 as an essential transcription factor in UTI, acting downstream of TLR4/TRAM (unpublished observation). To examine if Irf3 might be involved in TcpC mediated innate immune suppression, infection with CFT073 or *ΔTcpC* was established in Irf3 mutant mice (*Irf3^−/−^*), using wt C57BL/6 mice as controls. The difference in persistence between CFT073 and *ΔTcpC* in wild type mice was not observed during the early phase of infection in *Irf3^−/−^* mice (<day 5) (Figure 9A, B). Furthermore, in *Irf3^−/−^* mice, the early chemokine and neutrophil responses were delayed compared to responses in wt controls (Figure 9C, D). Significantly reduced responses to *ΔTcpC* were only observed from day five post-infection in *Irf3^−/−^* mice (Figure 9C, D), showing that the response kinetics differed from wt mice. Abscess formation in response to CFT073 was as frequent in the *Irf3^−/−^* as in wt mice (Figure 9E, F). The results suggest that the effect of TcpC on bacterial persistence and on the MIP-2 response are attenuated during the early phase of infection in IRF3-deficient mice and that IRF3 signaling is differentially activated depending on the TcpC status of the infecting strain (Please see figure 9B–D).

Taken together, the results show that bacterial TcpC modifies the innate host response broadly through inhibition of Tlr4, Myd88, Trif, IL-6/IL-1 and Irf3 dependent antibacterial effector functions.

## Discussion

Bacterial TIR-like proteins are important virulence factors, which act by inhibiting innate immunity, thus facilitating the survival and persistence of several pathogens. The *Salmonella enteritica* TlpA protein enhances bacterial survival in macrophages and mice [15] and the *Brucella* TcpB protein inhibits TIRAP mediated signaling and reduces systemic spread of bacteria during the early stages of infection [16]. The *E. coli* TcpC protein increases virulence in the urinary tract and we have previously proposed that TLR signaling is impeded through the MYD88 adaptor via direct binding of TcpC to MYD88 [12]. This study addressed the mechanism through which TcpC modifies the innate immune response in the infected host, by varying the innate immune genetic repertoire. The results show that TcpC requires Myd88 to act as a virulence factor *in vivo*. Transcriptomic analysis identified additional targets for TcpC in human cells, including the TRIF and IL-6 pathways, as well as IL-1α/β. *Tlr4^−/−^, Trif ^−/−^*, *Lps2^−/−^* and *Il-1β^−/−^* mice exhibited markedly different immune responses to TcpC stimulation and the TcpC dependent change in bacterial persistence and pathology was attenuated in these mice. The results thus suggest that pathways for host-defense can be fine-tuned by a bacterial virulence factor in order to paradoxically promote bacterial replication and pathology.

MyD88 was the first TLR adaptor to be identified and is shared by the TLRs as well as the interleukin-1 receptor [20], [21]. By targeting MyD88, TcpC would thus be expected to impair both TLR- and IL-1 dependent signaling pathways as well as related antibacterial effector functions. This interpretation was supported by the *in vivo* effects, which were not restricted to Myd88 but regulated by a group of genes encoding proteins with a TIR domain or regulated by such proteins. In *Myd88^−/−^* mice, essential chemokine and neutrophil responses to CFT073 infection were completely abrogated consistent with the importance of MyD88 for the overall innate immune response to these infections. The response to infection was strongly reduced also in *Tlr4^−/−^* mice confirming our previous findings that Tlr4 signaling is essential for the innate immune response to UTI and suggesting that that inhibition of TLR4 responses may be protective at the mucosal level [11], [22], [23], [24]. We have recently extended the analysis of potential eukaryotic interaction partners and have shown that the TIR-domain of TcpC binds to TLR4 in addition to MyD88 (unpublished observation). TcpC did not bind to TRIF or TLR2, however [12]. Thus, as expected by the differing aminoacid sequences, only selected TIR-domains bind TcpC. The difference in bacterial persistence, while reduced, was not abrogated in *Tlr4^−/−^* or *MyD88^−/−^* mice, however, possibly reflecting the involvement of pathways that remains intact in *Myd88^−/−^* mice. The interaction with TLR4 provides a molecular basis for inhibition of the TRIF-dependent arm of the TLR4 signaling cascade, thus possibly explaining, in part, the effects of TcpC in *Trif ^−/−^* mice.

This mode of action of TcpC was also supported by the experiments in murine macrophages, where responses to most of the TLR specific ligands were inhibited by purified TcpC-TIR protein. TcpC inhibited LPS-driven, MyD88 dependent and LPS-driven MyD88-independent TNF secretion, including TLR4-TRIF signaling. However, TcpC did not influence poly (I:C)-induced TNF-secretion, whether MyD88 dependent or not. The fact that poly (I:C)-induced TNF-secretion in the absence of TcpC was lower in MyD88-deficient cells compared to wild type cells cannot be interpreted to imply that poly (I:C) stimulates MyD88-dependent TNF-secretion, since resting MyD88-deficient macrophages are impaired in their basal expression of several cytokines including TNF [25]. Cytokines like TNF are in general harder to induce in MyD88-deficient cells, as also reported by Sun et al. [26]. In preliminary experiments, we have also analyzed the secretion of the chemokine KC and have not found differences in wild type or MyD88-deficient cells after stimulation with poly (I:C). Thus, poly (I:C) stimulates cells in a MyD88-independent but TRIF-dependent manner and the results are compatible with the *in vivo* effect.

TRIF has a well conserved TIR domain and several TRAF6 binding regions within the N- and C-terminal RIP homotypic interaction motifs (RHIM) [27], [28], [29]. TRAM is TLR4 specific [30], [31] and the myristoylated N-terminus associates to the plasma membrane and protein kinase Cε phosphorylation of Serine 6 and 16 is essential for TRAM activation [32], [33]. In contrast to MYD88, TRIF dependent signaling activates both NF-κB and IRF3/7 [30]. After TRAM dependent TLR4 activation, TRIF forms a complex with TRAF3, IRAK1 and an IKK-like kinase. TRAF family member associated NF-κB activator (TANK)-binding kinase 1 (TBK1) and the IKK homolog, IKKε, phosphorylate IRF3 at its C terminus [30], [34], initiating IFN-α/β transcription [29]. IRF7 becomes activated in a similar manner [4]. In this study, TcpC suppressed transcription of NF-κB and IRF7 as well as IL-8, TNF-α, IL-1α/β and IL-6, which is consistent with the effects on MYD88 and possibly TRIF. The *in vivo* response to infection supports the conclusion that TcpC also suppresses Trif dependent effector functions, however, as *Trif ^−/−^* mice have a functional Myd88 response, except for the cooperative TLR4 response to LPS [35]. The lack of pathology in infected *Trif ^−/−^* mice further suggests that Trif signaling is essential for efficient innate immune-mediated clearance of UPEC infection.

The transcriptomic analysis of infected human kidney cells suggested that TcpC also modifies proinflammatory cytokine signaling downstream of MYD88 and TRIF. The IL-6 pathway was strongly regulated and IL-1α/β expression was reduced by TcpC. The involvement of IL-1 was confirmed by infection of *Il-1β^−/−^* mice and the phenotype of the *Il-1β^−/−^* mice was quite convincing, as the difference in bacterial persistence between CFT073 and the TcpC deficient mutant in wt mice was abrogated in *Il-1β^−/−^* mice and the innate immune response to the two isogenic strains was similar. Bacterial clearance was rapid, further suggesting that IL-1 may be a significant factor in the mucosal response to UPEC and in the establishment of tissue pathology. The mechanism of TcpC modulation of IL-1 is not clear, however and may either relate to a TIR domain dependent interaction of TcpC with the IL-1 receptor, effects on upstream signaling involving Myd88 and Trif and the resulting IL-1 response or to other mechanisms.

Recently, Trif dependent innate immune responses have been shown to activate IRFs that regulate the transcription of pro-inflammatory genes, including IL-8, IL-6 and TNF, in addition to interferon genes [18]. In a parallel study, we have characterized a new TLR4/TRAM dependent pathway that mediates innate responses to P-fimbriated, uropathogenic *E. coli* through Irf3 (unpublished observation). Using a combination of transcriptomics, phosphorylation arrays and imaging technology, we detected TRAM phosphorylation, activation of MAP kinases including p38, CREB phosphorylation and nuclear IRF3 translocation. *Irf3^−/−^* mice lacking this pathway, developed rapid lethal kidney infections with extensive tissue damage and patients prone to acute pyelonephritis were shown to carry IRF3 promoter polymorphisms that reduce transcription efficiency. In the present study, interferon dependent pathways were differentially regulated by TcpC *in vitro* (data not shown) and in *Irf3^−/−^* mice the effects of TcpC were significantly delayed compared to wt mice. While this effect was transcient, the results further support the results suggesting that TcpC may modify the effects of the TRIF/Irf3 pathway and the progression to disease and pathology.

Innate immune activation by uropathogenic *E. coli* is orchestrated by specific virulence factors and essential aspects of the mucosal response show pathogen specificity [36]. Such interactions are needed, as innate immune responses are not activated by asymptomatic carrier strains in the epithelial tissue, which forms a barrier against interactions with inflammatory cells, with broader reactivity. For example, epithelial cells lack surface expressed CD14 and do not respond to conserved bacterial PAMPS like LPS [11], [36]. In addition, asymptomatic carrier strains may actively suppress the mucosal immune response, but the mechanisms are not fully understood. We have previously shown that pathogen specific TLR4 activation favours TRIF or MyD88, depending on the surface fimbriae, which are expressed in a pathogen specific manner and serve as crucial virulence factors involved in attachment and host tissue perturbation [11]. P fimbriated bacteria preferentially activate TLR4/TRIF signalling while Type 1 fimbriae trigger TLR4 responses mainly involving MyD88. The adaptor protein usage in infected host cells can even be shifted from TRIF to MyD88 by a change in fimbrial expression [11]. *E. coli* CFT073, used in the present study, expresses both P and type 1 fimbriae, thus activating TLR4 signalling involving TRIF and MyD88 responses in infected cells, providing a basis for independent targeting of these pathways by TcpC.

In conclusion, our results suggest that TcpC may act as a broad microbial innate immune response modulator *in vivo*, by preventing bacterial clearance and dysregulating inflammation, with destructive effects on infected tissues. This adds TcpC to an increasing number of components that regulate TLR and MYD88 dependent responses. On the host side, MYD88s inhibits the recruitment of IRAK-4, thus acting as a negative regulator of TLR signaling [37]. SIGIRR, IRAK-M, SOCS1, Triad3A and SARM, and the cytosolic domain of SIGIRR block TLR4 signaling through TIR-TIR interactions preventing the recruitment of IRAK and TRAF6 to MYD88. TcpC binds to TLR4 (unpublished data) and MYD88 [12] through TIR domain interactions and inhibits TLR4 and MYD88 dependent signaling *in vivo*, as well as downstream effector functions. While many molecular interactions remain to be defined, it is clear that this microbially induced suppression of the host defense differs, in the classical sense, from mucosal tolerance, which may be triggered by microbial or other mucosal antigens, but is defined by the involvement of specific immunity, with T cells as the main effector cells. In the case of TcpC, the innate immune response is modified and the “tolerant” state appears to result from active microbial inhibition, rather than from a lasting change in the immune status of the host, and from a direct modification of host resistance rather than by inducing tolerance. Further insights into these mechanisms may be helpful to distinguish “bad” from “good” inflammation and to understand how partial inhibition of MYD88, TRIF and TLR4 by TcpC results in pathology while complete gene deletion is protective. These findings also illustrate the importance of the host response as a generator of pathology and suggest the possibility of intervention to support “good” host responses, promoting bacterial clearance and tissue integrity while inhibiting pathology.

## Materials and Methods

### Ethics statement

C57BL/6 (wt), C57BL6/129 (wt), C57BL/6 *Tlr4^−/−^*, C57BL/6 *Myd88^−/−^*, C57BL/6 *Irf3^−/−^*, C57BL/6 *Il-1β^−/−^*, C57BL/6 *Trif ^Lps2/Lps2^* and C57BL6/129 *Trif ^−/−^* mice were bred and housed in the specific pathogen-free facilities of the MIG animal facilities (Lund, Sweden) with free access to food and water. All procedures were approved by the Animal Experimental Ethics Committee at the Lund District Court, Sweden (numbers M166-04 and M87-07), following Institutional, National, and European Union guidelines.

### Cell lines and reagents

The human epithelial cell line A498 (ATCC HTB44, human kidney carcinoma, Manassas, VA, USA) was cultured in RPMI-1640 supplemented with 1 mM sodium pyruvate, 1 mM non-essential amino acids, gentamycin (50 µg mL^−1^) and 5% fetal calf serum (PAA Laboratories, Pasching, Austria).

Alexa-fluor 568-goat anti-rabbit IgG and Alexa-fluor 488-goat anti-rat were from Invitrogen (Eugen, Oregon, USA). Anti-rat NIMP-R14 (ab2557) were from Abcam (Cambridge, UK) and goat normal serum were from Dako (Denmark). The reagents paraformaldehyde (Merck KGaA, Darmstadt, Germany), triton X-100 (VWR International Ltd, England), isopentane (Sigma-Aldrich, Germany), VECTASHIELD mounting medium (Vector Laboratories, USA), poly-L-lysine-coated glass slides (Thermo Scientific, Waltham, USA), DAPI (Invitrogen, Oregon, USA), tryptic soy agar (Difco, Detroit, USA) and hematoxylin-eosin stain (Histolab Products AB, Gothenburg, Sweden) were used. Mouse MIP-2 quantification kit was from R&D systems (Abingdon, UK). TargetAmp Nano-g Biotin-aRNA Labelling kit was from Epicentre Biotechnologies (Madison, USA), RNeasy clean-up from Qiagen (Alabama, USA) and the Procarta Human Cytokine 50-plex kit from Panomics (Fremont, USA).

### Mouse strains

*Tlr4^−/−^* [38], *Myd88^−/−^* [39], *Irf3^−/−^* [40] and *Il-1β^−/−^* [41] mice were generated in the C57BL/6 genetic background and *Trif ^−/−^* [28] mice were generated in the C57BL6/129 genetic background. Lps2, a non-functional codominant allele of Trif, was induced by N-ethyl-N-nitrosourea mutagenesis on a pure C57BL/6 mouse background [17] in the Scripps Institute animal facilities (La Jolla CA). *Il-1β^−/−^* mice were provided by Max Planck (Institute for Infection Biology, Berlin, Germany). Wild type C57BL6/129 mice are the 50% backcross of C57BL/6 mice. All the knock out mice (*Tlr4^−/−^*, *Myd88^−/−^*, *Irf3^−/−^* and *Il-1β^−/−^*) are backcross in 100% C57BL/6 wt mice and are pure.

### Bacterial strains

*E. coli* CFT073 was isolated from the blood and urine of a woman admitted to the University of Maryland Medical System for treatment of acute pyelonephritis [42]. This *hly*1+, *pap*1+, *sfa*1+ and *pil*1+ strain expresses P fimbriae, hemolysin, and Type 1 fimbriae and is highly virulent in the CBA mouse model of ascending UTI. It is cytotoxic for cultured human renal proximal tubular epithelial cells [43]. CFT073 expresses the TcpC protein and the *ectcp* sequences, which encode TcpC and were deleted in the *ectcp::kan* mutant (Δ*TcpC*), as described [12]. The strains were grown to stationary phase overnight on tryptic soy agar with appropriate selection and suspended in 10 ml of phosphate-buffered saline (PBS) (pH 7.2) to generate the bacterial suspension used for inoculation (10^9^ CFU, colony forming units/ml). The bacteria were tested for virulence factor expression, including P and Type 1 fimbriae [44].

### Experimental UTI mice model

Female C57BL/6, C57BL6/129, *Tlr4^−/−^*, *Myd88^−/−^*, *Trif ^−/−^*, *Trif ^Lps2/Lps2^*, *Il-1β^−/−^* and *Irf3^−/−^* mice were used at 8–12 weeks of age. *E. coli* urinary tract infection was established as described [45]. In brief, 0.1 ml of the bacterial suspension was administered into the bladders of anesthetized mice (10^9^ CFU/ml) with the help of a soft polyethylene catheter (inner diameter 0.28 mm, outer diameter 0.61 mm; Clay Adams, New Jersey USA). Prior to inoculation, urine samples were collected and cultured on blood agar plates to ensure that the mice were uninfected. After infection, urine was collected into sterile tubes through gentle pressure on the mouse's abdomen (5 h, 24 h and up to 6 days) and neutrophils were quantified by light microscopy using a Burker chamber. The urine samples and serial dilutions were quantitatively cultured on tryptic soy agar (TSA) plates. Mice were sacrificed by cervical dislocation while anesthetized. Bladders and kidneys were removed under sterile conditions, placed into a plastic bag containing 5 ml PBS (pH 7.2), homogenized in a Stomacher 80 homogenizer (Seward medical, London, UK) and plated on TSA plates. Subsequently, blood agar and TSA plates were incubated at 37°C overnight and visually scored for bacterial colonies. Kidneys were also prepared for hematoxylin-eosin staining or immunohistochemistry.

### Histology and immunohistochemistry

Kidneys were fixed in freshly prepared 4% paraformaldehyde soon after dissection and incubated overnight at 4°C. Further, the fixed tissues were incubated in 15% sucrose (4°C/24 hr) and washed in 25% ice cold sucrose (4°C/24 hr). Tissues were then frozen in isopentane at −40°C and stored at −80°C for further use. Cryostat sections were made with a steel knife (10 µm), mounted onto poly-L-lysine-coated glass slides and stained.

The kidney sections from all mouse groups were stained for dual immunohistochemistry as described [46]. Briefly, tissue sections were dried at 37°C for 15 min, washed in PBS-0.2% Triton X-100 (pH 7.2) (2×10 min/RT) and incubated (30 min/RT) with PBS-0.2% Triton X-100+ 5% goat normal serum (Dako, Denmark). Then the sections were incubated with primary anti-rat NIMP-R14 (ab2557, Abcam, Cambridge, UK; 1∶200) and antiserum to a synthetic peptide within the PapG adhesin (1∶200) for 2–3 hr at RT. Negative controls consisted of only normal goat serum (1∶200). The kidney sections were washed in PBS (3×5 min) and incubated (1 hr/RT) with secondary goat anti-rat immunoglobulins (1∶200), conjugated with Alexa 488 dye (A488; 495_ext_/519_em_ nm), and secondary goat anti-rabbit immunoglobulins (1∶200), conjugated with Alexa 568 dye (A568; 578_ext_/603_em_ nm), as fluorochrome (Invitrogen, USA). After washing in PBS (2×5 min), specimens were counterstained (3 min/RT) with DAPI (0.05 µM) for nuclei staining. Sections were washed again in PBS (10 min) and mounted with VECTASHIELD mounting medium (Vector Laboratories, USA) and kept in the dark. Sections were analyzed by fluorescence microscopy (AX60, Olympus Opticals, Hamburg, Germany) in the Department of Pathology, Lund University, Sweden.

### Cytokine measurements

Urine samples were collected at 0.6 hr and after 1, 2, 3, 4, and 6 days and stored at −20°C. MIP-2 in urine was quantified by ELISA using the Mouse MIP-2 quantification kit (R&D systems, Abingdon, UK) according to the manufacturer's instructions and urine was diluted five fold in sample buffer. The ELISA plates were read at 450 nm in a Labsystems Multiskan PLUS reader (Analytical Instruments LLC, Golden Valley, USA).

### DNA microarray analysis

For the microarray study, 350,000 A498 cells were seeded in 6-well plates and infected with CFT073 or Δ*TcpC* (10^8^ CFU/ml) upon confluency. Total RNA was extracted 4 hr after stimulation by acid guanidinium thiocyanate-phenol-chloroform extraction (Trizol, Invitrogen, USA) followed by a Qiagen RNeasy clean-up procedure. RNA was reverse-transcribed to double stranded cDNA and converted to biotin-labelled cRNA using a TargetAmp Nano-g Biotin-aRNA Labelling kit (Epicentre Biotechnologies, Madison, USA). Labelled cRNAs were hybridized onto an Illumina Human WG6v3 Expression Beadchip for 16 hours at 58°C. The arrays were then washed and stained based on the Illumina Wash Protocol and then scanned using a BeadArray Scanner 500GX. The background subtracted data were pre-processed to correct negative and non significant intensities. Pre-processed data was normalized using the cross correlation [47] and genes with a fold change of 2 were identified as differentially expressed. Data was preprocessed using RMA implemented in the free software packages R and Bioconductor (http://www.r-project.org). In identification of CFT073 or Δ*TcpC*-specific genes, fold change of ≥2 was used for the comparison CFT073 or Δ*TcpC* with its control and fold change of ≤1.5 was used for the comparison Δ*TcpC* or CFT073 with its control, respectively. In addition to the above fold change criteria, statistical *t*-test with *p*≤0.05 was further used in selection of differentially expressed genes or CFT073/Δ*TcpC* specific genes. To further study signaling pathways altered by TcpC, the differentially expressed genes were analyzed using the Ingenuity Pathway Analysis software with default settings (Ingenuity Systems, Redwood City, CA). In parallel, cDNA was also quantified by RT-PCR using human primers IL-6 (Hs00174131_m1), IL-8 (Hs00174103_m1), NFKB1 (Hs00231653_m1), cFOS (Hs01119266_g1), IRF3 (Hs01547283_m1), STAT1 (Hs01014002_m1), IRF7 (Hs00185375_m1) and TNF-α (Hs01113624_g1) from Applied Biosystems.

### Protein arrays

Kidney A498 cells were stimulated with CFT073 or Δ*TcpC* as described above. Culture supernatants were collected and cleared by centrifugation at 20,000×g before storage at −80°C. Cytokine profile analysis was performed in triplicate using the Procarta Human Cytokine 50-plex kit (Panomics, Fremont, USA) according to manufacturer's protocol. Cytokine levels were evaluated using a Luminex 100 system (Luminex, Austin, TX, USA).

### RT-PCR and ELISA of murine tubular kidney cells

Primary murine renal tubular epithelial cells (MRTEC) were harvested from murine kidneys (C57BL/6, *Trif ^Lps2/Lps2^*, *Myd88^−/−^*) following microdissection and brief collagenase digestion (2 mg/ml of Collagenase Type II). Cells were grown in hormonally defined RPMI supplemented with epidermal growth factor (Sigma, 50 pg/ml), insulin-transferrin-sodium selenite media supplement (Gibco, diluted 1∶100), Prostoglandin E_1_ (Sigma, 1.25 ng/ml), T3 (Sigma, 34 pg/ml), hydrocortisone (Sigma, 18 ng/ml), 10% heat-inactivated FCS, and 0.5 µg/ml gentamycin at 37°C. The cells were stimulated 5 days after the primary explantation at 100% confluence and infected with CFT073 or Δ*TcpC* (10^8^ CFU/ml) for 4 hrs. Total extracted mRNAs were converted to cDNA using RT2 First Strand Kit (SABioscience Corporation, Fredrick, MA, USA). The mouse primers used for RT-PCR GAPDH (QT01658692) and MIP-2 (QT00113253) were from Qiagen. Gene expression levels were calculated by the ΔCt method and normalized to house-keeping genes. cDNA was quantified by RT-PCR using a Rotor gene 2000 instrument (Corbett Life Science, Sydney, Australia) and normalized against GAPDH for each gene. In parallel, MIP-2 in culture supernatants was also quantified by ELISA using the Mouse MIP-2 quantification kit (R&D systems, Abingdon, UK) according to the manufacturer's instructions.

### BMDM and ligand stimulation assays

Wt or *Myd88^−/−^* BMDM (bone marrow derived macrophages) were cultured in 6-well plates at a concentration of 0.5×10^6^/well in DMEM supplemented with 10% FCS, 1% penicillin-streptomycin and 0.1% 2-mercaptoethanol. Cells were stimulated with the TLR ligands Pam3Cys, poly(I:C), LPS, flagellin or CpG-ODN 1826 in the absence or presence of titrated amounts of the purified recombinant TIR domain of TcpC (TIR-TcpC) for 3 h. TNF was quantified in the culture supernatant using ELISA Duo sets (R&D Systems) as described by the manufacturer.

### Statistical analysis

Bacterial counts and immune responses are presented as geomean ± SEM. Fisher's exact test and two-tailed T test was used for the analysis of bacterial counts and immune response. Student's t-test was used for protein array analysis. Wilcoxon's matched pairs test was used for paired comparisons. The level of significance was set at p<0.05 for all tests.

### List of ID numbers for genes and proteins of mouse and humans

Gene ID number for human TLR4 is 7099, mouse Tlr4 is 21898, human MYD88 is 4615, mouse Myd88 is 17874, human TRIF is 148022, mouse Trif is 106759, human IRF3 is 3661, mouse Irf3 is 54131, human IL-1B is 3553 and mouse Il-1b is 16176.

## Supporting Information

Figure S1 Response in murine tubular kidney cells infected with CFT073 and Δ*TcpC*. MIP-2 response of murine tubular cells from wt, *Myd88^−/−^* or *Trif ^Lps2/Lps2^* mutant mice infected with CFT073 or Δ*TcpC*(Means ± SEMs of three experiments). P values (**p<0.01 for CFT073 versus Δ*TcpC* mutant and p<0.0001 comparing wt and *Trif ^Lps2/Lps2^* versus *Myd88^−/−^* mutant cells.
(0.60 MB PDF)

Table S1 Comparison of gene expression levels in pathways regulated by CFT073 and the Δ*TcpC* mutant. The Wilcoxon matched pairs test was used to compare the effect of different types of stimulation on different gene classes.
(0.11 MB PDF)
