# Supplementary figures and images for: Inhibition of TIR Domain Signaling by TcpC: MyD88-Dependent and Independent Effects on Escherichia coli Virulence
Source: PLoS Pathog. 2010 Sep 23;6(9):e1001120. doi: 10.1371/journal.ppat.1001120 (PMC2944809; doi:10.1371/journal.ppat.1001120)

Figure S1

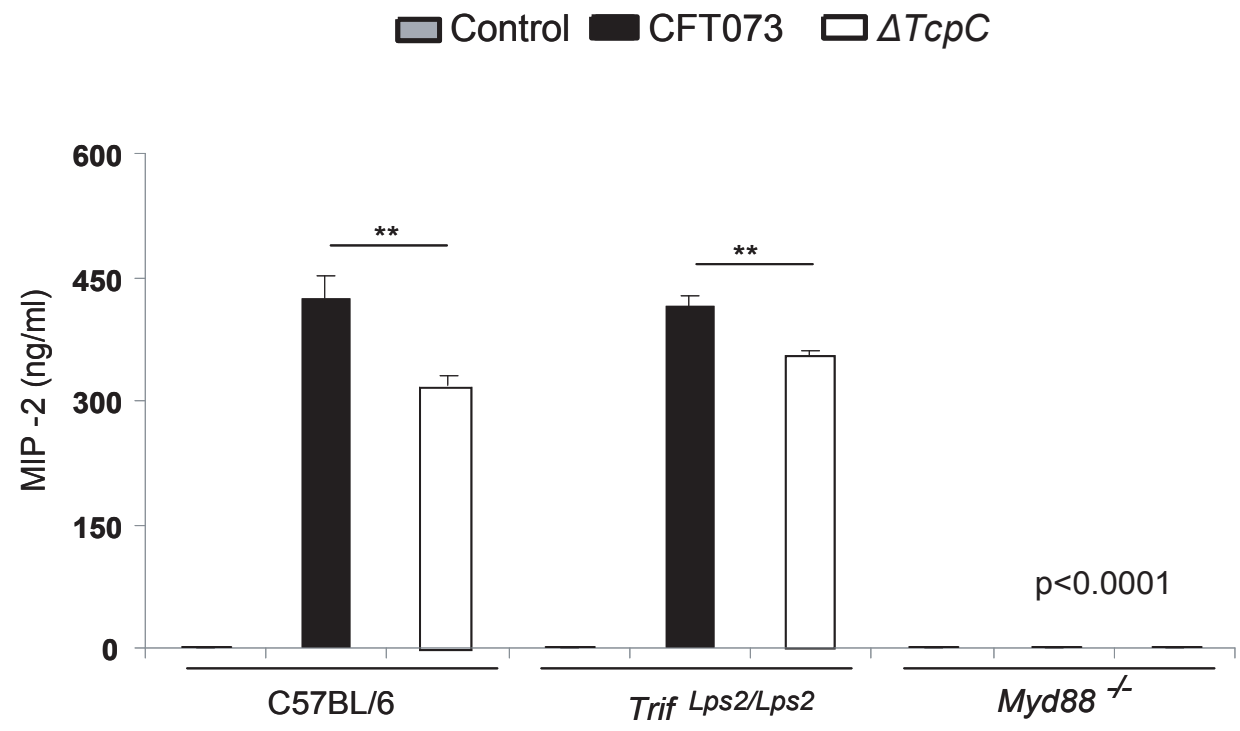

Supplement: Figure S1 — Response in murine tubular kidney cells infected with CFT073 and ΔTcpC. MIP-2 response of murine tubular cells from wt, Myd88−/− or Trif Lps2/Lps2 mutant mice infected with CFT073 or ΔTcpC(Means ± SEMs of three experiments). P values (**p<0.01 for CFT073 versus ΔTcpC mutant and p<0.0001 comparing wt and Trif Lps2/Lps2 versus Myd88−/− mutant cells. (0.60 MB PDF) [file ppat.1001120.s001.pdf]
