# Supplementary material for: Inhibition of TIR Domain Signaling by TcpC: MyD88-Dependent and Independent Effects on Escherichia coli Virulence
Source: PLoS Pathog. 2010 Sep 23;6(9):e1001120. doi: 10.1371/journal.ppat.1001120 (PMC2944809; doi:10.1371/journal.ppat.1001120)
Supplement: Table S1 — Comparison of gene expression levels in pathways regulated by CFT073 and the ΔTcpC mutant. The Wilcoxon matched pairs test was used to compare the effect of different types of stimulation on different gene classes. (0.11 MB PDF) [file ppat.1001120.s002.pdf]

Table S1. Comparison of gene expression levels in pathways regulated by CFT073 and the ΔTcpC mutant.

Functional Classification	Entrez Gene ID	Gene Symbol	Gene Title	Fold Change Over Control CFT073	*ΔTcpC*	**p values*
**Pattern recognition**						
a) MyD88 dependent	3569	IL6	Interleukin 6	16.982	21.303	0.001
	7124	TNF	Tumor necrosis factor (TNF superfamily, member 2) (TNF), mRNA.	8.128	9.266	0.01
	3665	IRF7	Interferon regulatory factor 7	2.675	3.543	0.03
	4790	NFKB1	Nuclear factor of kappa light polypeptide gene enhancer in B-cells 1	3.794	4.269	0.05
	4791	NFKB2	Nuclear factor of kappa light polypeptide gene enhancer in B-cells 2	4.401	4.509	ns
	3592	IL12A	interleukin 12A	4.958	5.035	ns
	10333	TLR6	Toll-like receptor 6	2.015	1.909	nt
	5970	RELA	v-rel reticuloendotheliosis viral oncogene homolog A	2.966	2.633	ns
b) TRIF dependent	3659	IRF1	Interferon regulatory factor 1	9.480	9.659	ns
	3660	IRF2	Interferon regulatory factor 2	2.286	2.148	nt
	3665	IRF7	Interferon regulatory factor 7	2.675	3.543	0.03
	4790	NFKB1	Nuclear factor of kappa light polypeptide gene enhancer in B-cells 1	3.794	4.269	0.05
	4791	NFKB2	Nuclear factor of kappa light polypeptide gene enhancer in B-cells 2	4.401	4.509	ns
	3456	IFNB1	Interferon, beta 1	24.915	12.843	0.001
	7096	TLR1	Toll-like receptor 1	2.139	2.136	ns
	148022	TICAM1	Toll-like receptor adaptor molecule 1	2.472	2.261	ns
	5970	RELA	v-rel reticuloendotheliosis viral oncogene homolog A	2.966	2.633	ns
	6352	CCL5	Chemokine (C-C motif) ligand 5	4.392	3.412	0.01
c) Complement activation	5806	PTX3	Pentraxin-related gene, rapidly induced by IL-1 beta	36.656	44.911	0.01
	718	C3	Complement component 3	2.419	2.032	ns
	5293	PIK3CD	Phosphoinositide-3-kinase, catalytic, delta polypeptide	2.251	2.109	ns
**IL-1/IL-6 signaling ** **						
a) Activators	3569	IL6	Interleukin 6	16.982	21.303	0.001
	3576	IL8	Interleukin 8	12.397	12.826	ns
	3552	IL1A	Interleukin 1, alpha	7.690	10.468	0.03
	3553	IL1B	Interleukin 1, beta	10.651	13.296	0.02
	7124	TNF	Tumor necrosis factor (TNF superfamily, member 2) (TNF), mRNA.	8.128	9.266	0.01
	7130	TNFAIP6	Tumor necrosis factor, alpha-induced protein 6	3.050	3.627	ns
	4982	TNFRSF11B	Tumor necrosis factor receptor superfamily, member 11b	1.456	2.308	0.02
	4790	NFKB1	Nuclear factor of kappa light polypeptide gene enhancer in B-cells 1	3.794	4.269	0.05
	4791	NFKB2	Nuclear factor of kappa light polypeptide gene enhancer in B-cells 2	4.401	4.509	ns
	3725	JUN	Jun oncogene (JUN)	2.103	1.431	0.05
	2354	FOSB	FBJ murine osteosarcoma viral oncogene homolog B	3.184	0.326	0.001
	6722	SRF	serum response factor(c-fos serum response element-binding transcription facto	2.162	1.948	nt
	1051	CEBPB	CCAAT/enhancer binding protein (C/EBP), beta	2.414	2.507	ns
	5606	MAP2K3	Mitogen-activated protein kinase kinase 3	3.436	2.758	ns
	5608	MAP2K6	Mitogen-activated protein kinase kinase 6	0.269	0.288	ns
	5970	RELA	v-rel reticuloendotheliosis viral oncogene homolog A	2.966	2.633	ns
b) Inhibitors	3557	IL1RN	Interleukin 1 receptor antagonist	2.214	1.898	nt
	8651	SOCS1	Suppressor of cytokine signaling 1	3.157	3.752	ns
	4792	NFKBIA	Nuclear factor of kappa light polypeptide gene enhancer in B-cells inhibitor, α	7.484	6.778	0.05
	4793	NFKBIB	Nuclear factor of kappa light polypeptide gene enhancer in B-cells inhibitor, β	2.262	2.019	nt
	4794	NFKBIE	Nuclear factor of kappa light polypeptide gene enhancer in B-cells inhibitor, Є	7.245	7.906	ns
	64332	NFKBIZ	Nuclear factor of kappa light polypeptide gene enhancer in B-cells inhibitor, ζ	3.690	2.423	0.01
	9641	IKBKE	Inhibitor of kappa light polypeptide gene enhancer in B-cells, kinase epsilon	2.011	2.298	ns

**** - Significant difference between CFT073 versus ΔTcpC infected cells. Remaining comparisions were not significant (ns) or not testable (nt)***

***** - IL-1/IL-6 signalling pathways were significantly regulated (p<0.026).***
